# The MATTERHORN trial: Percutaneous MitraClip vs surgical repair in heart-failure-related secondary mitral regurgitation

**DOI:** 10.21542/gcsp.2025.2

**Published:** 2025-02-28

**Authors:** Susy Kotit

**Affiliations:** Aswan Heart Centre (AHC), Aswan, Egypt

## Abstract

**Introduction:** Functional mitral regurgitation (FMR) is a common complication of heart failure and is associated with poor prognosis and increased morbidity and mortality. Surgical mitral valve repair (MVRe) and MitraClip transcatheter edge-to-edge repair (M-TEER) are used to improve FMR, however, there has not been sufficient comparison between the benefits of both approaches. The MATTERHORN trial aimed to compare the efficacy and safety outcomes of percutaneous versus surgical repair in high-risk patients with heart failure and FMR, to determine the noninferiority of transcatheter edge-to-edge therapy in this population.

**Study and results:** 208 patients were randomized in a 1:1 ratio to either M-TEER with MitraClip device or surgical mitral valve repair or replacement. The average age was 70.5 ±  7.9 years, 39.9% were female, mean LVEF was 43.0 ±  11.7%, 85.7% of patients had NYHA class III or IV, 96.0% had Mitral regurgitation grade ≥3, 38.2% had grade 4+, median effective regurgitant orifice area was 0.22cm^2^ and the mean regurgitant fraction was 57.0 ±  21.0%. Successful repair was seen in 96.1% in the M-TEER group and 98.6% in the surgery group. At 1 year, at least one primary endpoint event (death, hospitalization for heart failure, mitral valve re-intervention, implantation of an assist device, or stroke) occurred in 16.7% of the M-TEER group and 22.5% of the surgery group. All-cause death occurred in 8.3% and 10.3% of patients, respectively. Recurrence of MR grade ≥3 at 1 year and mitral valve reintervention was 8.9% and 5% in the M-TEER group and 1.5% and 2% in the surgical group, respectively. Overall, 41.0% of the patients in the intervention group and 77.3% in the surgery group had at least one adverse event, of which 35% and 66%, respectively, were serious.

**Lessons learned:** Transcatheter edge-to-edge repair demonstrated noninferiority to surgery for a composite of death, rehospitalization for heart failure, stroke, reintervention, or assist device implantation at 1-year. M-TEER exhibited significantly fewer major adverse events within 30 days post-procedure, albeit with a slightly higher MR recurrence rate at one year. This study underscores the potential of M-TEER as a less-invasive, yet effective, alternative to surgery for FMR, mitigating surgical risks and offering a faster recovery path, especially for patients deemed unsuitable for surgery. However, long-term data is crucial for guiding policies.

## Introduction

Functional mitral regurgitation (FMR) resulting from left ventricular (LV) dysfunction, which impairs leaflet coaptation of a structurally normal mitral valve (MV), is a common complication of heart failure (HF), with moderate to severe mitral regurgitation (MR) affecting 24% to 59% of these patients^2–5^. FMR is associated with adverse outcomes, including increased morbidity and mortality^1, 6, 7^, aggravated by the underlying cardiac pathology. The one-year mortality rates for FMR typically range from 15–40%^5, 8–10^ and the advantages of mitral valve intervention remain uncertain^1^.

Medical management remains pivotal in FMR treatment^3, 11^, especially in advanced right or left HF, where the outcomes of MV interventions may offer limited benefits^12, 13^. Surgical mitral valve repair is recommended for suitable candidates according to current guidelines. In contrast, percutaneous approaches, notably MitraClip transcatheter edge-to-edge repair (M-TEER), are advised for patients at high surgical risk or with unfavorable anatomical considerations^11, 14^.

Although MV surgery can alleviate symptoms^19–21^, it is associated with operative risks and uncertain long-term benefits. Its impact on altering the natural progression of the primary disease (cardiomyopathy/LV dysfunction) or enhancing survival remains inconclusive^15–18^.

M-TEER has emerged as an alternative to surgery. The MitraClip device, a polyester-covered cobalt-chromium clip, is inserted *via* the femoral vein and guided into the left atrium (LA) through transseptal puncture under fluoroscopic and transesophageal echocardiography (TEE) guidance, which mimics the surgical Alfieri stitch by grasping and reducing MR-causing mitral leaflet edges ^1^. This procedure has been shown to improve symptoms^22–28^, enhance survival rates^22–28^, reduce heart failure-related hospitalizations, and enhance quality of life for select patients unresponsive to medical therapy ^1^.

However, there has not been a sufficient comparison between the benefits of surgery and M-TEER in HF-related FMR. The goal of the Multicenter Mitral Valve Reconstruction for Advanced Insufficiency of Functional or Ischemic Origin (MATTERHORN) trial was to compare the efficacy and safety outcomes of M-TEER *versus* surgical mitral valve repair in high-risk patients with heart failure and FMR and to determine the non-inferiority of transcatheter edge-to-edge therapy in this specific population.

## Methods

The MATTERHORN trial was a multicenter, prospective, randomized, open-label, non-inferiority trial that compared the MitraClip system to reconstructive mitral valve surgery in the setting of depressed left ventricular ejection fraction (LVEF) and functional MR to determine whether transcatheter edge-to-edge therapy is non-inferior to mitral valve surgery in high-risk patients who are at high surgical risk.

Inclusion in the trial required clinically significant functional MR (grade 3+ or 4+) which was defined by meeting at least two of the following criteria: effective regurgitant orifice area of at least 20 mm 2 , biplane vena contracta width of more than eight mm, a regurgitant volume of at least 30 ml, a regurgitant fraction of at least 50%, or at least two hospitalizations for acute heart failure during the 12 months before enrolment, left ventricular ejection fraction (LVEF) ≥ 20%, symptoms of heart failure (New York Heart Association [NYHA] class ≥ 2) despite guideline-directed therapy, and eligibility for both transcatheter edge-to-edge repair or mitral-valve surgery as determined by the local heart team.

**Figure 1.**
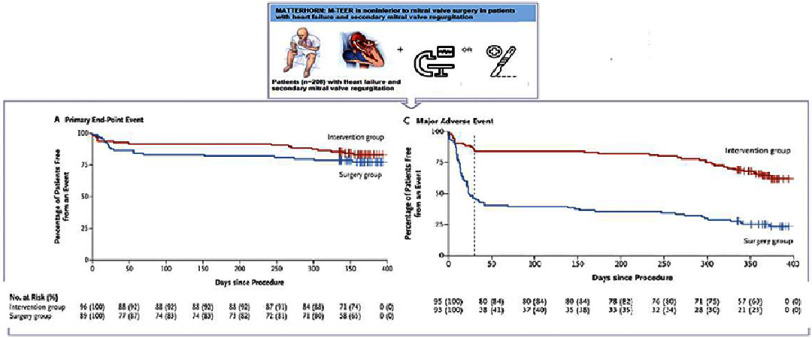
MATTERHORN trial.

Patients were randomized in a 1:1 ratio to either M-TEER with MitraClip device (Abbott Vascular) or surgical mitral valve repair or replacement (Figure 1). For mitral valve surgery, surgical access and technique, including concomitant ablation procedures or tricuspid valve surgery, were left to the discretion of the surgeon. Procedures were performed according to local best practice.

Patients were evaluated at baseline, at hospital discharge, 30 days and 1 year after the procedure. Standardized transesophageal and transthoracic echocardiographic examinations were performed before the procedure, before discharge, and 12 months after the procedure.

Key exclusion criteria included severe tricuspid regurgitation, other concomitant severe valve disorders, and coronary revascularization or cardiac resynchronization device implantation within 1 month prior to enrolment.

The primary efficacy endpoint was the composite of death from any cause, hospitalization for heart failure, mitral valve re-intervention, left ventricular assist device implantation, or stroke within 1 year after the procedure.

The key secondary efficacy endpoint was the recurrence of MR grade ≥3 (mitral regurgitation grade 3+ (moderate-to-severe disease) or 4+ (severe disease) at 1 year.

Additional secondary endpoints included the change in the 6-minute walk distance, New York Heart Association functional class, and Minnesota Living with Heart Failure Questionnaire score.

The primary composite safety endpoint assessed at 30 days included death (all-cause), myocardial infarction, major bleeding, stroke or transient ischemic attack, rehospitalization (all-cause), all reintervention or nonelective cardiovascular surgery, renal failure, deep wound infection, mechanical ventilation >48 h, gastrointestinal complications requiring surgery, new-onset atrial fibrillation (AF), septicemia, and endocarditis.

## Results

A total of 208 patients were randomized, with 104 patients allocated to each study arm.

The average age was 70.5 ± 7.9 years and 39.9% were female. The mean LVEF was 43.0 ± 11.7%, and heart failure was NYHA class III or IV in 85.7% of patients.

Mitral regurgitation grade ≥ 3 was present in 96.0% of the patients (*n* = 191), of which 38.2% was grade 4+. Median effective regurgitant orifice area was 0.22 cm^2^ (0.17 to 0.28); and the mean regurgitant fraction was 57.0 ± 21.0%.

The median Society of Thoracic Surgeons Predicted Risk of Mortality score (range of scores, 0 to 100%, with higher scores indicating a greater risk of death within 30 days after the procedure) was 2.0 (interquartile range, 1.1 to 3.7), the median European System for Cardiac Operative Risk Evaluation (EuroSCORE) II score (range of scores, 0 to 100%, with higher scores indicating a greater risk of in-hospital death) was 3.0 (interquartile range.7 to 4.3), and 13.1% of the patients had received previous cardiac resynchronization therapy.

In the intervention group, transcatheter edge-to-edge repair was successful and resulted in a mitral regurgitation grade of ≤2 in 96.1% of patients at 1 year, 48.5% of patients received one device and 40.6% received two devices.

In the surgical group, 72.0% underwent mitral valve repair, and 28.0% underwent mitral valve replacement. Mitral surgery resulted in a mitral regurgitation grade of ≤2 in 98.6% of patients at 1 year.

At hospital discharge, the percentage of patients receiving a renin–angiotensin–aldosterone system inhibitor and the percentage receiving triple heart failure therapy was 81.4% in the intervention group and 57.9% in the surgery group.

### Primary endpoint

A total of 96 patients (92.3%) in the intervention group and 89 (85.6%) in the surgery group had data available at 1 year. The median follow-up time was 370 days (range: 366–374 days) in the intervention group and 368 days (range: 364–372 days) in the surgery group.

At 1 year, at least one primary endpoint event—death, hospitalization for heart failure, mitral valve re-intervention, implantation of an assist device, or stroke—occurred in 16.7% of the M-TEER group and in 22.5% of the surgery group (estimated mean difference, −6 percentage points; 95% confidence interval [CI], −17 to 6; *P* < 0.001 for non-inferiority). The relative risk ratio was 0.74 (95% CI, 0.41 to 1.34). All-cause death occurred in 8.3% and 10.3% of patients in the M-TEER and surgery groups, respectively.

Mitral valve reintervention occurred in 5% of the M-TEER group and 2% of the surgical group, while implantation of an LV-assist device was required in 2% and 4% of patients, respectively. Stroke occurred in 1% of the M-TEER group and 4% of the surgical group.

### Secondary endpoints

Recurrence of MR grade ≥ 3 at 1 year occurred in 8.9% of patients in the TEER group *vs.* 1.5% in the surgical group (estimated mean difference, 7 percentage points; 95% CI, 0 to 14; *P* = 0.02 for noninferiority).

The median change in the 6-minute walk distance from baseline to 1 year was 31 m (−56 to 85) in the intervention group and 32 m (−51 to 80) in the surgery group. The linear regression model adjusted for site and 6-minute walk distance at baseline yielded a regression coefficient of 5.22 (95% CI, −42.39 to 52.82), with the surgery group as the reference group (R 2 = 0.318). This confidence interval remained above −50 m, meeting the non-inferiority criterion.

At 1 year, 23.3% of the patients in the intervention group and 18.2% in the surgery group had New York Heart Association functional class III or IV heart failure, compared to 82.4% and 89.1% at baseline, respectively.

The median change in the Minnesota Living with Heart Failure Questionnaire (range of scores, 0–105, with higher scores indicating lower quality of life) at 1 year was −10 (−20 to 0) in the intervention group and −5 (−19 to 4) in the surgical group. In-hospital staya after intervention were 4 days and 12 days in the M-TEER and surgery groups, respectively.

### Safety and adverse events

The safety endpoint, which was a composite of major adverse events within 365 (± 30) days after the procedure, occurred in 36.5% of patients in the intervention group and 75.3% in the surgery group (estimated mean difference, −39 percentage points; 95% CI, −51 to −25).

Major events during intervention occurred in three patients, including intraprocedural partial detachment of the clip in one patient and chordae rupture in two patients leading to valve replacement. Reinterventions were needed in eight patients in the surgery group, three of which were related to thoracic bleeding and four to deep wound infections.

At 30 days, 2.0% of the patients in the intervention group and 4.3% of those in the surgery group died. Major bleeding (as defined by the Valve Academic Research Consortium (VARC)) occurred in 3.1% of patients in the intervention group and 24.4% in the surgery group (estimated difference, −21 percentage points; 95% CI, −31 to −12). New-onset atrial fibrillation occurred in 3.1% of the patients in the intervention group and 27.8% in the surgery group (estimated difference, −25 percentage points; 95% CI, −35 to −15). Stroke did not occur in the patients in the intervention group at 30 days, in contrast to the 4.4% occurrence rate in the surgery group.

Overall, 41.0% and 77.3% of the patients in the intervention and surgery groups, respectively, experienced at least one adverse event. Serious adverse events occurred in 35.0% and 66.0% of patients in the intervention and surgery groups, respectively.

## Discussion

The MATTERHORN trial is the first randomized trial to directly compare mitral transcatheter edge-to-edge repair (M-TEER) with surgery in patients with heart failure and functional mitral regurgitation eligible for mitral valve surgery, showing noninferiority of M-TEER in this patient cohort.

At one-year, the study revealed at least one primary endpoint event (death, heart failure hospitalization, mitral valve re-intervention, assist device implantation, or stroke) in 16.7% of the patients in the M-TEER group, in contrast to 22.5% in the surgery group.

While the surgical group experienced higher rates of all-cause mortality, LV-assisted device implantation, and stroke, mitral valve reintervention was more prevalent in the M-TEER group (5% *vs.* 2%). The recurrence of moderate-to-severe MR was notably higher in the TEER group (8.9% *vs.* 1.5%), with a 3% crossover to surgery within 12 months among TEER patients.

Both groups exhibited improvements in NYHA functional class, with the surgical group showing greater symptom enhancement and the intervention group displaying more favorable scores on the Minnesota Living with Heart Failure Questionnaire. The in-hospital stay after the intervention was longer in the surgical group.

A composite of major adverse events, including surgical bleeding, new-onset atrial fibrillation, and stroke, which are common postoperative sequelae, occurred more often in the surgery group.

Although both treatments effectively reduced MR severity, the M-TEER group demonstrated lower rates of procedural complications and faster recovery, offering substantial advantages, particularly for high-risk surgical patients. The MATTERHORN trial demonstrated the non-inferiority of mitral TEER compared to surgery for the composite endpoint at one year, with a favorable safety profile in heart failure patients with FMR, consistent with previous research showing reduced HF hospitalizations and mortality rates^29–31^.

This trial highlights M-TEER as a secure and efficient alternative to surgery for individuals with heart failure and functional mitral regurgitation, with a superior safety profile and non-inferior efficacy compared with conventional surgical methods, particularly beneficial for elderly populations and those unsuitable for surgery.

However, it is important to note that 1 year of follow-up is insufficient to determine the long-term outcomes of intervention or surgery, and extended follow-up is essential for both groups. As the patients were enrolled in the trial for more than seven years, a longer follow-up time should have been available. The noninferiority margin of 17.5% used in this study was also deemed too high. Furthermore, the loss to follow-up and consent withdrawal were significant, possibly affecting the results.

It is also essential to determine whether intervention (M-TEER or surgery) improves disease prognosis, as most of these patients have very poor survival due to ventricular disease and more than 70% die at 5 years, regardless of the procedure ^1^. Therefore, it is crucial to compare the results presented in this study to those of a medical therapy arm, especially since almost half of the cohort had an LVEF greater than 40%, to determine the benefits and clinical significance of both strategies.

## Conclusion

In patients with heart failure and functional MR, transcatheter edge-to-edge repair demonstrated non-inferiority to surgery for a composite of death, rehospitalization for heart failure, stroke, reintervention, or assist device implantation at one year.

M-TEER exhibited significantly fewer major adverse events within 30 days post-procedure, albeit with a slightly higher MR recurrence rate at one year. This study highlights the potential of M-TEER as a less invasive yet effective alternative to surgery for functional mitral regurgitation, mitigating surgical risks, and offering faster recovery, especially in patients deemed unsuitable for surgery. However, long-term data is crucial for guiding policies.

## References

[1] Asgar AW, Mack MJ, Stone GW (2015). Secondary mitral regurgitation in heart failure: Pathophysiology, prognosis, and therapeutic considerations. J Am Coll Cardiol.

[2] Robbins JD, Maniar PB, Cotts W, Parker MA, Bonow RO, Gheorghiade M (2003). Prevalence and severity of mitral regurgitation in chronic systolic heart failure. Am J Cardiol.

[3] Bartko PE, Hülsmann M, Hung J (2020). Secondary valve regurgitation in patients with heart failure with preserved ejection fraction, heart failure with mid-range ejection fraction, and heart failure with reduced ejection fraction. Eur Heart J.

[4] Chioncel O, Lainscak M, Seferovic PM (2017). Epidemiology and one-year outcomes in patients with chronic heart failure and preserved, mid-range and reduced ejection fraction: an analysis of the ESC Heart Failure Long-Term Registry. Eur J Heart Fail.

[5] Rossi A, Dini FL, Faggiano P (2011). Independent prognostic value of functional mitral regurgitation in patients with heart failure. A quantitative analysis of 1256 patients with ischaemic and non-ischaemic dilated cardiomyopathy. Heart.

[6] Coats AJS, Anker SD, Baumbach A (2021). The management of secondary mitral regurgitation in patients with heart failure: a joint position statement from the Heart Failure Association (HFA), European Association of Cardiovascular Imaging (EACVI), European Heart Rhythm Association (EHRA), and Eu. Eur Heart J.

[7] Camaj A, Thourani VH, Gillam LD, Stone GW (2023). Heart failure and secondary mitral regurgitation: A contemporary review. J Soc Cardiovasc Angiogr Interv.

[8] Perez de Isla L, Zamorano J, Quezada M (2006). Prognostic significance of functional mitral regurgitation after a first non-ST-segment elevation acute coronary syndrome. Eur Heart J.

[9] Hickey MS, Smith LR, Muhlbaier LH (1988). Current prognosis of ischemic mitral regurgitation. Implications for future management. Circulation.

[10] Lamas GA, Mitchell GF, Flaker GC (1997). Clinical significance of mitral regurgitation after acute myocardial infarction. Survival and Ventricular Enlargement Investigators. Circulation.

[11] Vahanian A, Beyersdorf F, Praz F (2021). ESC/EACTS Guidelines for the management of valvular heart disease. Eur Heart J.

[12] Adamo M, Fiorelli F, Melica B (2021). COAPT-like profile predicts long-term outcomes in patients with secondary mitral regurgitation undergoing MitraClip implantation. JACC Cardiovasc Interv.

[13] Iliadis C, Metze C, Körber MI, Baldus S, Pfister R (2020). Impact of COAPT trial exclusion criteria in real-world patients undergoing transcatheter mitral valve repair. Int J Cardiol.

[14] Otto CM, Nishimura RA, Bonow RO (2020). ACC/AHA guideline for the management of patients with valvular heart disease: A report of the American College of Cardiology/American Heart Association Joint Committee on Clinical Practice Guidelines. Circulation.

[15] Wu AH, Aaronson KD, Bolling SF, Pagani FD, Welch K, Koelling TM (2005). Impact of mitral valve annuloplasty on mortality risk in patients with mitral regurgitation and left ventricular systolic dysfunction. J Am Coll Cardiol.

[16] Silberman S, Oren A, Klutstein MW (2006). Does mitral valve intervention have an impact on late survival in ischemic cardiomyopathy. Isr Med Assoc J.

[17] Calafiore AM, Iacò AL, Gallina S, Al-Amri H, Penco M, DiMauro M (2013). Surgical treatment of functional mitral regurgitation. Int J Cardiol.

[18] Milano CA, Daneshmand MA, Rankin JS (2008). Survival prognosis and surgical management of ischemic mitral regurgitation. Ann Thorac Surg.

[19] Acker MA, Parides MK, Perrault LP (2014). Mitral-valve repair versus replacement for severe ischemic mitral regurgitation. N Engl J Med.

[20] Acker MA, Jessup M, Bolling SF (2011). Mitral valve repair in heart failure: Five-year follow-up from the mitral valve replacement stratum of the Acorn randomized trial. J Thorac Cardiovasc Surg.

[21] Shah AS, Hannish SA, Milano CA, Glower DD (2005). Isolated mitral valve repair in patients with depressed left ventricular function. Ann Thorac Surg.

[22] Stone GW, Lindenfeld J, Abraham WT (2018). Transcatheter mitral-valve repair in patients with heart failure. N Engl J Med.

[23] von Bardeleben RS, Rogers JH, Mahoney P (2023). Real-world outcomes of fourth-generation mitral transcatheter repair: 30-day results from EXPAND G4. JACC Cardiovasc Interv.

[24] Cox ZL, Zalawadiya SK, Simonato M (2023). Guideline-directed medical therapy tolerability in patients with heart failure and mitral regurgitation: The COAPT trial. JACC Heart Fail.

[25] Rubbio APopolo, Testa L, Grasso C (2022). Transcatheter edge-to-edge mitral valve repair in atrial functional mitral regurgitation: Insights from the multi-center MITRA-TUNE registry. Int J Cardiol.

[26] Webb JG, Hensey M, Szerlip M (2020). 1-Year outcomes for transcatheter repair in patients with mitral regurgitation from the CLASP study. JACC Cardiovasc Interv.

[27] Kang DH, Park SJ, Shin SH (2019). Angiotensin receptor neprilysin inhibitor for functional mitral regurgitation. Circulation.

[28] Milwidsky A, Mathai SV, Topilsky Y, Jorde UP (2022). Medical therapy for functional mitral regurgitation. Circ Heart Fail.

[29] SG W, JoAnn L, AW T (2018). Transcatheter mitral-valve repair in patients with heart failure. N Engl J Med.

[30] SG W, AW T, JoAnn L (2023). Five-year follow-up after transcatheter repair of secondary mitral regurgitation. N Engl J Med.

[31] Asgar AW, Tang GHL, Rogers JH (2024). Evaluating mitral TEER in the management of moderate secondary mitral regurgitation among heart failure patients. JACC Heart Fail..

